# Pathology and Treatment of Asthma

**Published:** 1864-10

**Authors:** Hyde Salter

**Affiliations:** Lecturer on Physiology and Pathology at the Charing-Cross Hospital Medical School, and Assistant-Physician to the Hospital


### PATHOLOGY AND TREATMENT OF ASTHMA.

By HYDE SALTER, M.D., F.R.S., F.R.C.P.,
Lecturer on Physiology and Pathology at the Charing-Cross Hospital Medical School, and Assistant-Physician to the Hospital.

### II. ON THE TREATMENT OF ASTHMA BY THE IODIDE OF POTASSIUM.

This is a remedy that, in the opinion of many competent men, holds a high place in the treatment of asthma. Dr. Williams evidently thinks highly of it. I see many asthmatic patients who have been under the care of that eminent and able physician, and I find that for almost all of them he has ordered iodide of potassium. I remember some years ago receiving a long and interesting letter from my friend Dr. Oke, of Southampton, begging me to try it, and assuring me that he found it an almost unfailing remedy, and had seen it succeed in the most obstinate cases; indeed, he regarded it in the light of a specific. In many other and equally respectable directions I have heard its praises as loudly sounded.

But I must say that, according to my own experience, it does not deserve so high a place as has been given it. I find it entirely fail in a great many cases, while those in which its success is complete are comparatively few. I do not believe that in one case in five it of sufficient permanent advantage to be worth persevering in. Still, one case in five would be a great deal in such a disease as asthma; a disease so painful, and often so intractable; and I should not think it right to omit its trial in any case in which it had not been fairly tried. How to explain the lower estimate I have formed of its value than others have I cannot tell; but the very frequency with which I see it in the prescriptions of other physicians tells, I think, against it; for if it had done any material good—it had been a success —why should the patients come to me, and not rather continue under the care of those at whose hands they were receiving benefit? The frequency with which it is found in prescriptions for asthma represents, I think, the general opinion that is entertained on the part of those who prescribe it, rather than its utility to those who take it.

Sometimes, however, I have seen most striking results attend its use, as the following cases will show:—

E. H——, a lady aged thirty, who had suffered from asthma for seven years past, came under my care in September, 1863. Her paroxisms were of two kinds—very severe ones, lasting several days, at long intervals; and slight ones, occurring every night, and disturbing her sleep for an hour or two. From these last she had been suffering every night for some weeks when I saw her. Omitting many of the details of her case, I may mention the following as the most relevant:—Diet appears to exercise no influence on her attacks. Damp places agree with her the worst, and she is never well for the first day or two on going to a new place. She is liable to what she calls attacks of renal congestion, in which the urine is very thick and high-colored, and she thinks that this state of the urine is associated with and produces the asthma. Her father is a martyr to rheumatic gout, quite crippled by it, and has been for years; an uncle died of gout quite young. She has tried an infinity of medicines, and, as far as their effect go, they may be divided into two classes; those which give her slight relief, and those which give her no relief at all. Among the former are, inhaling chloroform, smoking stramonium, smoking various forms of cigarettes, burning nitre paper and inhaling the fumes, ipecacuanha as an emetic, mustard plasters, blisters, chloric ether in thirty-minim doses. Among the latter are, strychnine and nux vomica, valerian, lobelia, hot strong coffee, sulphuric ether, Indian hemp. The benefit derived from inhaling chloroform, fumes of nitre paper, from ipecacuanha as an emetic, and from chloric ether, is great at the time, for the smaller attacks, but in each case evanescent.

When I first saw this lady, she was staying at Chertsey, and having the miner attacks every night. I prescribed for her extract of stramonium, and one or two remedies which she had not tried. She called on me again about the 5th of October, and informed me that what I had prescribed for her did not seem to have affected her in any way; she still had the slight attacks every night. She was going away in three days to a place in Surrey, where she had always been bad and had had some of her most violent attacks; indeed, she had never been there without being violently asthmatic the whole time, and she looked forward to her visit with great apprehension. The house to which she was going was built, as she described it, almost in a well—in a place surrounded with water on all sides, and which was rather *wet* and *damp*. I ordered her five grains of iodide of potassium and twenty minims of aromatic spirit of ammonia, in a wineglass of water, three times a day. I saw her husband on the 22d of October, seventeen days afterwards, and his report was as follows:—She had begun the iodide of potassium a day or two after I had ordered it, and had not had an attack of any kind, severe or light, since. The minor attacks had entirely ceased, and she slept uninterruptedly through the night, a thing she had not done for two or three months: she had gone to the dreaded place, and no attack had occurred—the first time in her life that that had ever happened. Her husband did not know when he had seen her so well. She was daily gaining flesh and strength. The lady herself, with great simplicity, gave the strongest possible testimony to the effect of the remedy by saying, in her written account of herself, that "she had been so well since she had been taking it that she had had no opportunity of trying what its effects would be upon her asthma." To which of course I replied, that I did not care how long the same result should keep her ignorant of the virtues of the remedy.

It is now nearly a month since she has been taking the iodide, and she still remains perfectly free from her former symptoms. Occurring, as the change did, suddenly, and coincidently with the taking of the medicine, and under the most unfavorable circumstances—that is, when she was going to a place where she had never before escaped severe asthma as long as she was in it, I cannot but attribute the result to the remedy. What will be the effect of leaving it off, and whether on future occasions its results will be equally striking, the future only will show.*

The following case, in which the iodide appeared to be equally beneficial, was under my care during the past autumn:—

T. H——, a tall, pallid, spare man, aged sixty-two, had had asthma for six years, and for the last three the attacks had been frequent and very severe. He generally had an attack once a week, and if he escaped a fortnight thought himself very lucky. When I first saw him he had had for some time slight attacks every morning, about four o'clock, that woke him from sleep, and compelled him to sit up and cough and wheeze for an hour. There was no history of gout in the case; but there was clear evidence of occasional attacks of bronchitis. With regard to

* About a month after writing the above, I heard this lady had had a severe attack of acute bronchitis from exposure to cold. She was taking the iodide of potassium at the time. The bronchitis was very severe, so that for a day or two her life was in danger; but she had *no asthma*, although on all former occasions on which she had had bronchitis it had induced asthma. On the abatement of the bronchitis, I advised the resumption of the iodide of potassium; and at the time I last heard from her there had been no reappearance of the asthma, and this was fully two months from the time it was first given.

treatment, there was the old story; an infinity of remedies had been tried, and, with a single exception, nothing had done him any permanent good. That exception was chloroform, which never failed to give immediate relief. On waking each morning with his usual attack, from half a drachm to a drachm was inhaled. The difficulty of breathing at once subsided, the patient went off into a tranquil sleep, and there was an end of it; whereas, if the chloroform was not given, the dyspnœa would go on increasing, become very tedious, and very likely culminate in a regular attack. But with this single exception, all remedies that had been tried appeared inert. Several things that had not been employed I made a trial of, but with an equally unfavorable result till I tried iodide of potassium. The effect of this was very soon shown. No severe attack occurred after it was commenced, and in a few days the regular morning attacks ceased also. The patient now slept all night without disturbance, and there was no longer any necessity for resorting to the chloroform. This went on for six weeks; the iodide of potassium was then left off. In a few days the asthma began to show itself again, and in a week or two was as bad as ever. The iodide was then resumed, with the same beneficial results as before. I have not heard of this patient now for more than a month; and this very circumstance inclines me to hope that this remedy still keeps his enemy at bay.

It should always be borne in mind, in giving iodide of potassium for asthma, that it is often some time before it begins to take effect. I have a patient at the present time under my care who has been taking it for three weeks past in eight-grain doses three times a day, but it is only during the last week that any decided improvement has taken place in him. He has lost his spasms; the expectoration has very much decreased; and he has ceased to experience an abiding "thickness" and tightness of breathing that he had in the intervals of the attacks, and which never left him. His nurse tells me that whereas before, for months past, whenever he was asleep his breathing was audible and labored, and accompanied with a slight wheezing, it is now inaudible and tranquil. Yet for the first fortnight this patient derived no apparent benefit whatever from the drug, and was anxious to give it up; now, however, he is convinced of the good it is doing him, and is anxious to continue it. It may be asked, Why do I think that the improvement is really to be assigned to a remedy that seems to remain so long inoperative? Why may not the apparent benefit be a coincidence, and the drug be really doing him no good whatever? I think the improvement is the work of the iodide, for two reasons. In the first place, from the fixedness of the patient's previous condition for a great length of time, no medicines or any other agencies that were brought to bear upon him making any difference in him. In the second place, from this tardiness of the action of the iodide of potassium corresponding with its action in other affections. How long is it, for instance, before it makes any appreciable impression upon a goître, however complete and satisfactory its results may ultimately be!

I used to think that the benefit derived from iodide of potassium in asthma was entirely due to its beneficial influence in chronic bronchitis, and therefore that the only cases of asthma in which it did any good were cases in which chronic bronchitis and asthma coëxisted, and the one was the existing cause of the other. I am compelled, however, now to abandon that view; for in some of the cases in which its efficacy has been the most striking there has not been a trace of bronchitis.

Another theory that I once held I am also obliged to abandon—namely, that it was of advantage only in those cases in which the asthma was due to a gouty or rheumatic-gouty condition; and that it was by relieving this condition that it relieved the consequent asthma. In two of the cases that I have related this view would be borne out, for there was evidence of gout in both of them; but in the third there was not a trace. Moreover, I have seen cases of true gouty asthma in which iodide of potassium has been of no service.

Of its ultimate and exact *modus operandi* I can neither offer any explanation nor form any reasonable opinion. I am not, however, the less satisfied of its occasional great value, and of the propriety of its use in any case in which it has not been tried.—*London Lancet.*

